# Definition and management of odontogenic maxillary sinusitis

**DOI:** 10.1186/s40902-019-0196-2

**Published:** 2019-03-29

**Authors:** Soung Min Kim

**Affiliations:** 1 Oral and Maxillofacial Microvascular Reconstruction LAB, Ghana Health Service, Brong Ahafo Regional Hospital, P.O. Box 27, Sunyani, Brong Ahafo Ghana; 2 Department of Oral and Maxillofacial Surgery, Dental Research Institute, School of Dentistry, Seoul National University, 101 Daehak-ro, Jongno-gu, Seoul, 110-768 South Korea

**Keywords:** Rhinosinusitis, Odontogenic maxillary sinusitis (OMS), Functional endoscopic sinus surgery (FESS), Modified endoscopy-assisted maxillary sinus surgery (MESS), Odontogenic infection

## Abstract

**Background:**

Maxillary sinusitis of odontogenic origin, also known as maxillary sinusitis of dental origin or odontogenic maxillary sinusitis (OMS), is a common disease in dental, otorhinolaryngologic, allergic, general, and maxillofacial contexts. Despite being a well-known disease entity, many cases are referred to otorhinolaryngologists by both doctors and dentists. Thus, early detection and initial diagnosis often fail to detect its odontogenic origin.

**Main body:**

We searched recent databases including MEDLINE (PubMed), Embase, and the Cochrane Library using keyword combinations of “odontogenic,” “odontogenic infection,” “dental origin,” “tooth origin,” “sinusitis,” “maxillary sinus,” “maxillary sinusitis,” “odontogenic maxillary sinusitis,” “Caldwell Luc Procedure (CLP),” “rhinosinusitis,” “functional endoscopic sinus surgery (FESS),” “modified endoscopy-assisted maxillary sinus surgery (MESS),” and “paranasal sinus.” Aside from the PRISMA (Preferred Reporting Items for Systematic reviews and Meta-Analyses) trial, there have been very few randomized controlled trials examining OMS. We summarized the resulting data based on our diverse clinical experiences.

**Conclusion:**

To promote the most efficient and accurate management of OMS, this article summarizes the clinical features of rhinosinusitis compared with OMS and the pathogenesis, microbiology, diagnosis, and results of prompt consolidated management of OMS that prevent anticipated complications. The true origin of odontogenic infections is also reviewed.

## Background

Maxillary sinusitis of odontogenic or dental origin, also known as chronic maxillary sinusitis of dental origin, or odontogenic maxillary sinusitis (OMS), is a comparatively well-known disease in dental, otorhinolaryngologic, and allergic contexts. Any diseases arising from dental or dentoalveolar structures could affect the Schneiderian membrane (SM), leading to diverse pathologic disease presentations in the maxillary sinus. Exact and accurate diagnosis of odontogenic origin is necessary to avoid the long-term administration of inappropriate medications or unnecessary surgical management.

The aim of this article is to provide information about the pathophysiology of OMS for comparisons with chronic or acute maxillary sinusitis, including chronic rhinosinusitis (CRS) and acute bacterial rhinosinusitis (ABRS). Clinical features including the pathogenesis and microbiology of OMS are reviewed, and appropriate management with accurate diagnosis, prompt consolidated treatment, and prevention of anticipated complications is summarized.

## Main text

We conducted a search of recent, up-to-date databases including MEDLINE (PubMed), Embase, the Cochrane Library, and other online tools using keyword combinations of “odontogenic,” “odontogenic infection,” “dental origin,” “tooth origin,” “sinusitis,” “maxillary sinus,” “maxillary sinusitis,” “odontogenic maxillary sinusitis,” “Caldwell Luc procedure (CLP),” “rhinosinusitis,” “functional endoscopic sinus surgery (FESS),” “modified endoscopy-assisted maxillary sinus surgery (MESS),” and “paranasal sinus.” The results are summarized based on our diverse clinical experiences.

A statement of ethics approval was provided by the Department of Oral and Maxillofacial Surgery at Seoul National University Dental Hospital, with the approval of the Institutional Review Board of Seoul National University (S-D20170005).

### Chronic and acute rhinosinusitis

The importance of appropriate diagnosis and management of chronic or acute rhinosinusitis cannot be emphasized enough, because nasal or sinus problems including nasal stuffiness, nasal airway obstruction, nasal drainage, and postnasal drip are very common [1–3].

#### Classification of rhinosinusitis

Rhinitis has been confused with rhinosinusitis and described using terminology that is a more accurate definition for describing inflammations of the inner nasal cavity involving the paranasal sinuses. A diagnosis of rhinosinusitis requires two of the following symptoms: nasal obstruction, middle facial pain, mucopurulent discharge, and decreased smell, with additional observation of mucosal inflammation required for final consolidated diagnosis. The treatment of rhinosinusitis varies according to etiology, and initial differentiation between acute and chronic forms should be made while considering the patient’s previous history, present symptoms, and the results of nasal endoscopic examination or careful intraoral inspection. Evidence-based therapy may be initially managed by physicians in cases of acute or chronic rhinosinusitis, and more difficult symptoms that are refractory to avoidance can be referred to an allergist for further immunotherapy [4]. Acute rhinosinusitis (ARS) is defined according to symptom duration as follows: infectious ABRS, with purulent nasal discharges, obstruction, and pain with sensation of fullness within 4 weeks; subacute rhinosinusitis (SRS) between 4 and 8 weeks; and CRS with symptoms lasting more than 8 weeks despite treatment with medications. Rhinosinusitis has been also classified into allergic or non-allergic, occupational, and other types of rhinitis syndromes.

#### Mucociliary clearance functions of SM

**Fig. 1:**
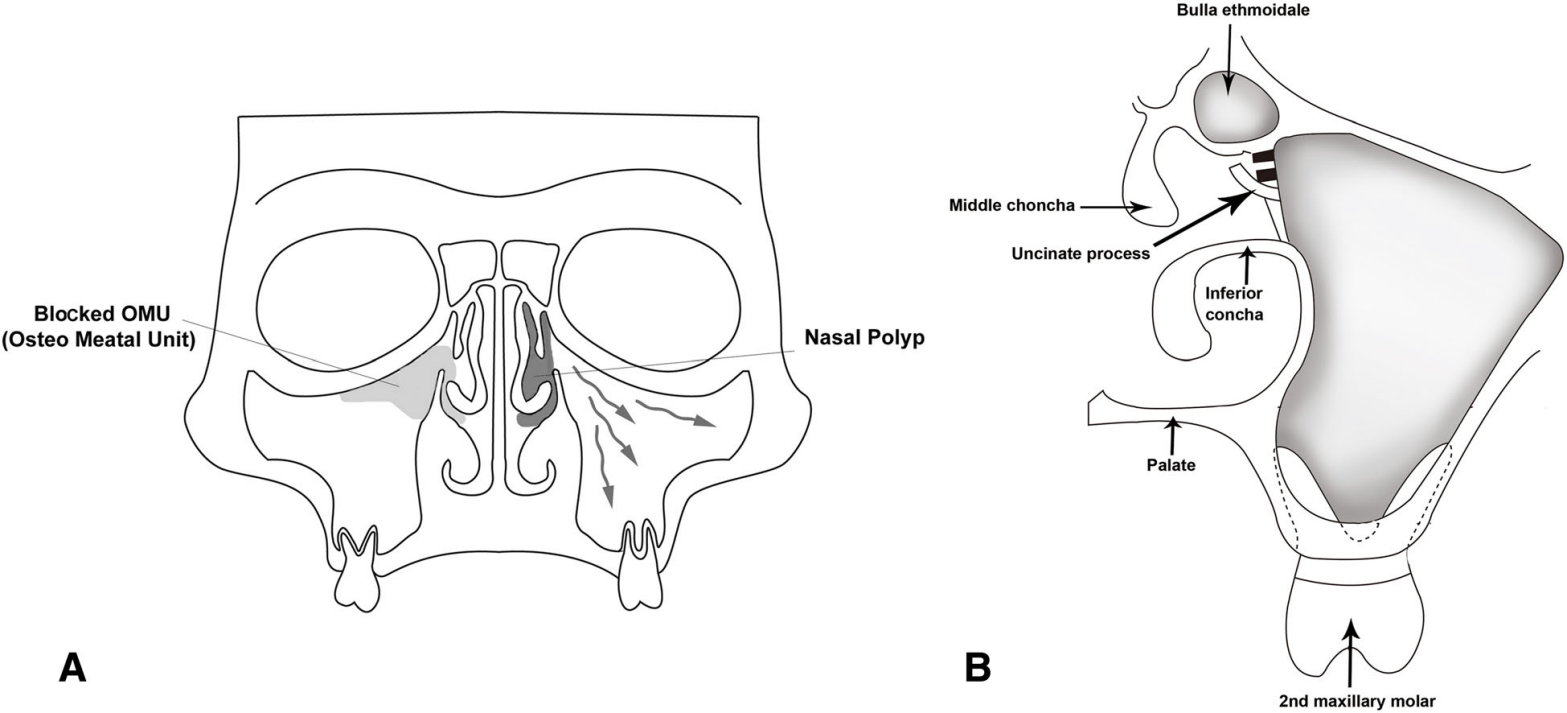
A coronal schematic representation of the posterior maxillary teeth with the sinonasal complex. Normal mucociliary clearance activity through the maxillary sinus to the osteo-meatal unit is shown as arrows, representative antrochoanal polyps on the superior and medial sinus walls appear dark, and ethmoidal polyps appear as gray asterisks (**a**). The osteo-meatal unit showing the middle and inferior nasal conchae, uncinate process, and bulla ethmoidale (**b**)

The pseudostratified ciliated columnar epithelium, known as SM, lines the inner respiratory mucosa of the maxillary sinuses. The SM produces mucus that moves to the ostium for drainage into the nasal cavity against normal gravity, with movement of cilia around the maxillary sinus occurring in a synchronized pattern (Fig. 1). This mucus, passing from the nasal cavity to the nasopharynx, is swallowed and passes into the esophagus and stomach. Any interruption of these basic movements of mucus by reduced ciliary activity or obstruction of ostia can result in sinus disease and symptoms. Each ostium of the anterior ethmoidal sinus, frontal sinus, and maxillary sinus is closely approximated in the middle nasal meatus, and together, these comprise the osteo-meatal unit (OMU). Thus, any inflammation or blockage of the OMU will induce sinusitis, including cases involving several sinuses, referred to as pan-sinusitis.

**Fig. 2:**
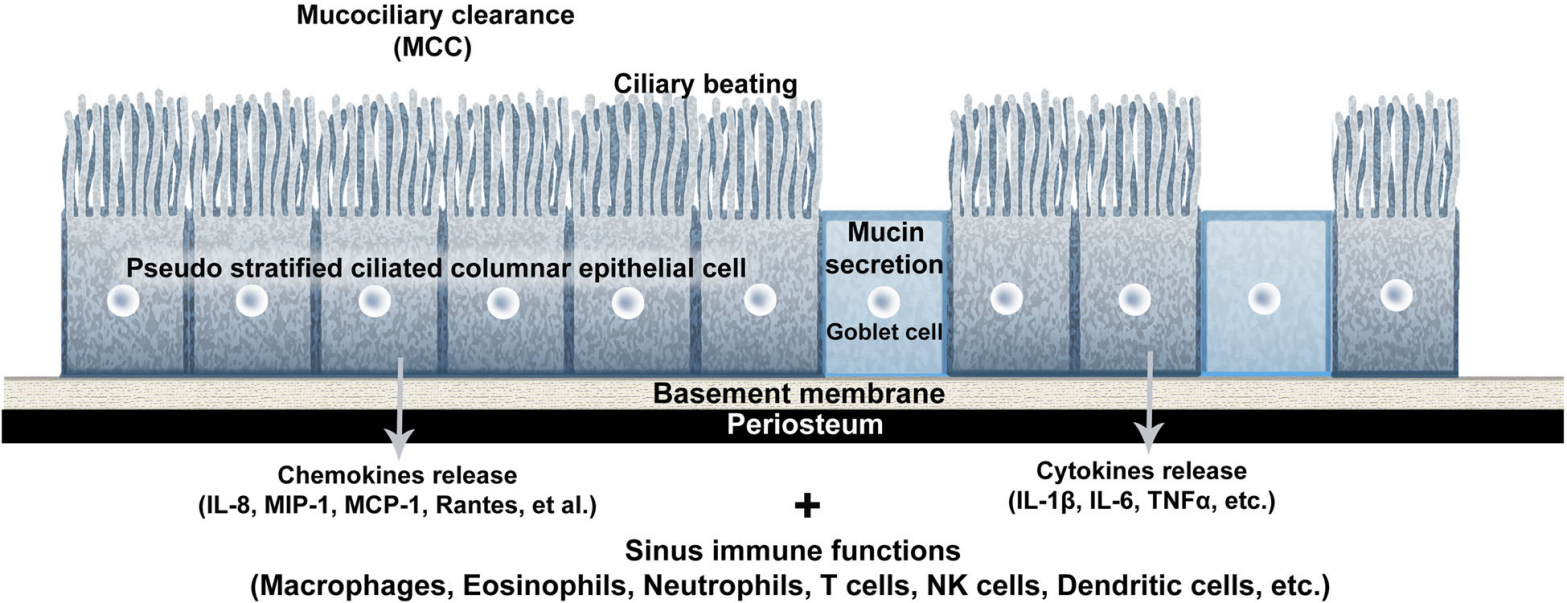
Schematic drawing of immunity-related mucociliary clearance in the pseudostratified ciliated columnar epithelium of the maxillary sinus. Abbreviations: MCC mucociliary clearance, MCP-1 monocyte chemotactic protein 1, MIP-1 macrophage inflammatory protein-1, IL interleukin, TNF tumor necrosis factor

The epithelial cells of SM play essential roles in mucociliary clearance (MCC) and keeping the upper airway clean by driving continuous ciliary beating to move inhaled foreign bodies, bacteria, fungi, and viruses toward the oropharyngeal airway. These basic protective functions are aided by the airway epithelium with mucin secretions that create ion or fluid transport to maintain mucous viscosity. Several chemokines are secreted according to pathogen exposure levels to activate inflammatory or protective immune pathways by recruitment of macrophages, dendritic cells, eosinophils, neutrophils, T cells, and NK cells (Fig. 2) [4, 5]. Several cytokines, including IL-1β, IL-6, TNFα, IL-8, and monocyte chemotactic protein 1, are also released. These epithelial cells of SM are connected by tight junctions to form a physical defensive wall, and mucociliary transport is managed by the formation of reactive oxygen and nitrogen species through control of antimicrobial peptides such as lactotransferrin, lysozyme, and defensins (Fig. 2).

#### Definition and course of ABRS

The main etiology of ABRS is infection by native bacteria in ambient air through the upper aerodigestive tract. Thus, the first goal of ABRS management is reducing bacterial infections accompanied by symptomatic improvement. Most cases of inflammatory sinusitis, including ABRS, occur within 7 to 10 days after upper respiratory tract viral infection. ABRS patients can recover from viral infections but may have continuous symptoms such as facial pain and nasal congestion with rhinorrhea. Nasal or sinus stasis may occur because of reduced MCC activity and the high anatomical position of the ostium. Sometimes ethmoidal or antrochoanal polyps, known anatomical variations, aggravate stasis by blocking physiologic drainage through the middle nasal meatus (Fig. 1). Due to the variation in pathophysiologic entities in OMS, exact management of the original dental problems should follow ABRS symptom relief. Amoxicillin can usually be administered to ABRS patients according to individual clinical progress and comorbidities, while computed tomographic scans are helpful for the objective diagnosis of complicated symptoms or other severe complications such as intracranial extension [6].

#### Definition and course of CRS

Typical CRS is defined as having more than two of the following symptoms for more than 12 weeks: facial pressure pain, decreased smell, nasal congestion, rhinorrhea, or postnasal drip. Facial pressure pain is usually described as a dull and localizing pressure pain in the upper cheek with continuous headache on the same side of the forehead. Decreased smell can be divided into partial hyposomia and total anosmia, which are both related to anterior ethmoidal mucosal opacifications. Sometimes such patients complain of reduced taste sensation, known as ageusia. Nasal congestion is also expressed as nasal stiffness or fullness and nasal cavity blockage. Anterior or posterior rhinorrhea is defined as a thick yellow or brown mucus discharge, which is more common in ABRS than in CRS patients.

CRS etiology is associated with diverse anatomical variations and inhalation of foreign bodies. Cigarette smoking or allergic rhinitis is also known to influence CRS, along with social economic status. Anatomical variations, such as deviated septum or middle nasal turbinate, and abnormal Haller cell size or agger nasi cells, can cause obstruction of the OMU and consequently induction of CRS. Several known environmental air irritants, such as sulfur dioxide, ozone, and formaldehyde, have also been shown to impact MCC function. Allergic rhinosinusitis has underlying genetic or immune factors related to the development of CRS [4, 5].

CRS is classified as CRS without polyp or allergic fungal sinusitis according to polyposis or fungal infections. More recently, the pathogenesis of CRS has been shown to involve immune responses changes. Polyps or cystic fibrosis results in abnormal changes of the sinonasal epithelium that alter MCC function [5–7]. Inhalation exposure to irritants, such as bacteria including *Staphylococcus aureus* (*S. aureus*), fungi, viruses, and proteases, degrades the functions of respiratory epithelial barriers. Dysregulated epithelial cells may release inflammatory molecules such as thymic stromal lymphopoietin, which can aggravate the development of type 2 immune inducer responses in CRS in nasal polyp patients. Innate immune cells such as type II innate lymphoid, mast cells, and eosinophils are increased, and these cells can release type 2 cytokines including IL-4, IL-5, and IL-13 that further perpetuate the ongoing inflammatory response [5–7]. In contrast, adaptive immune cells such as dendritic, T helper type 2, native B, and activated plasma cells are also increased in CRS with nasal polyps, and thus contribute to increased local production of antibodies within the sinonasal tissue [6, 7]. Type 2 cytokines are also thought to contribute to decreased tissue plasminogen activator and increased Factor XIIIa levels, which in turn lead to increased fibrin deposition and cross-linking within nasal polyps (Fig. 2).

Treatment of CRS is based upon severity and etiology. Corticosteroids and additional antibiotics can be helpful when coupled with saline irrigation through the nasal cavity. Discrimination between diverse causes of CRS while ruling out other symptoms is essential to ensure good outcomes after CRS management [8]. However, all avenues of clinical management for CRS result in limited outcomes because of the heterogeneous pathology of CRS.

### Odontogenic maxillary sinusitis

The incidence of OMS has likely been underreported, with 10–12% of OMS cases attributed to odontogenic infections [9–12] in the otorhinolaryngological literature. Studies that are more recent suggest a much higher incidence than previously reported, with chronic maxillary sinusitis (CMS) comprising 30–40% of all cases of CMS [13].

#### Development and growth of maxillary sinus

The maxillary sinus may occasionally be absent or hypoplastic during development and show growth spurts at 0–3 and 7–12 years, which correspond with the development and eruption of the permanent dentition and pubertal facial growth [14, 15]. A pneumatization process continues with maxillary sinus growth throughout the whole lifetime, until the sinus floor is at a level below the nasal floor after loss of the involved teeth. The first and second molars are close to the inferior wall of the maxillary sinus, with the premolar teeth less so and ectopic canine teeth only occasionally adjacent. The OMU is located superiorly on the medial wall and average 2.4 mm in diameter, while the bony window is much larger [14–16]. The effective opening of the ostium may be reduced by the projection of the uncinate process, which is an extension of the inferior turbinate and the surrounding soft tissues (Fig. 1).

**Fig. 3:**
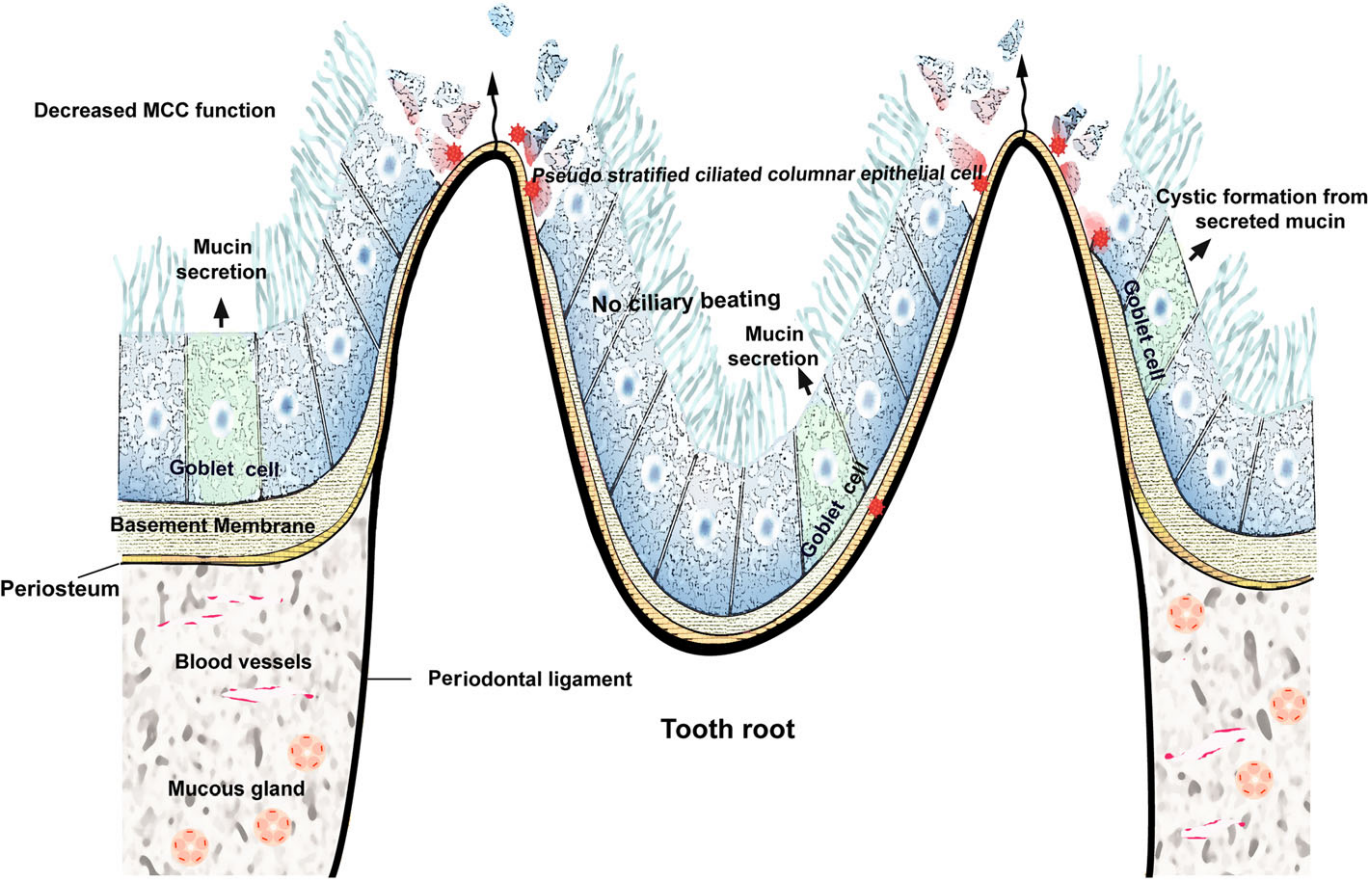
Schematic drawing of the breakdown of mucociliary clearance (MCC) function due to odontogenic infection. The dysregulated epithelial mucosal barrier is widened according to infection severity and duration

The bony wall that separates the maxillary sinus from the dental roots varies, ranging from complete loss in which the roots are covered only by SM to a thickness of more than 12 mm. The mean distance between the maxillary molar and premolar roots and the maxillary sinus is 1.97 mm, which suggests that the tips of the roots might project into the floor of the sinus, causing small elevations or prominences along the SM [17, 18]. These intimate anatomical relations of the upper molar teeth to the maxillary sinus facilitate the development of periapical or periodontal odontogenic infection inside the maxillary sinus [19] (Fig. 3).

#### Definition and etiology of OMS

**Fig. 4:**
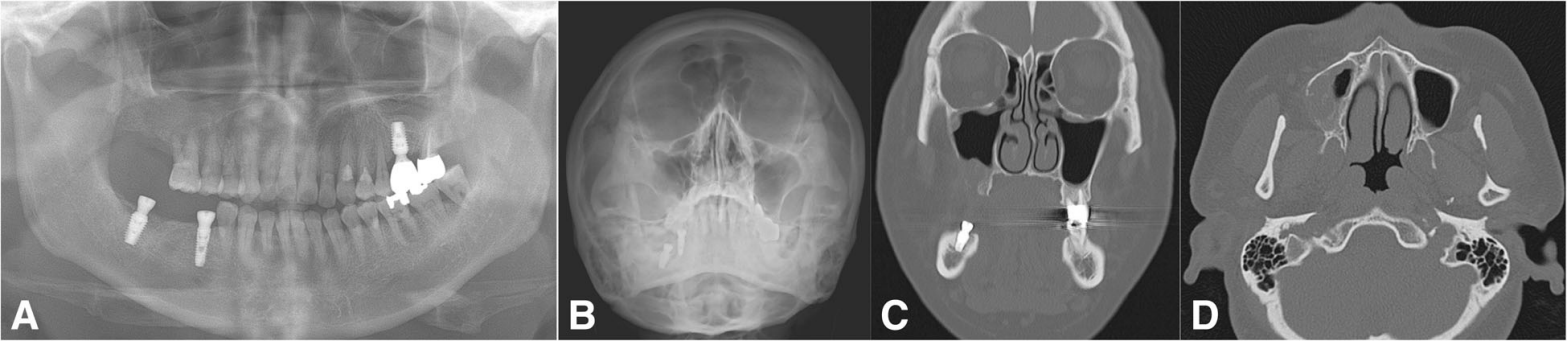
Chronic oronasal fistula after second molar extraction with several points of alveolar bony resorption indicates odontogenic maxillary sinusitis. Preoperative panoramic (**a**), Water’s (**b**), coronal CT scan (**c**), and axial CT scan (**d**) views

**Fig. 5:**
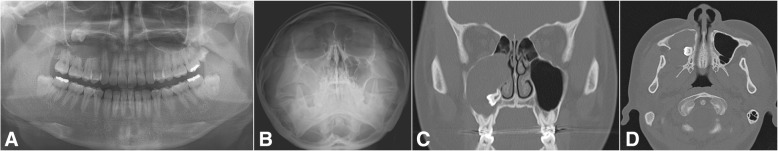
A case of odontogenic maxillary sinusitis originating from a tooth bearing a huge cyst in the right maxillary sinus. Preoperative panoramic view (**a**), Water’s view (**b**), coronal CT scan view showing a bony expansible cystic mass with ostium obstruction (**c**), and axial CT scan view showing the posterior expansional mass (**d**)

**Fig. 6:**
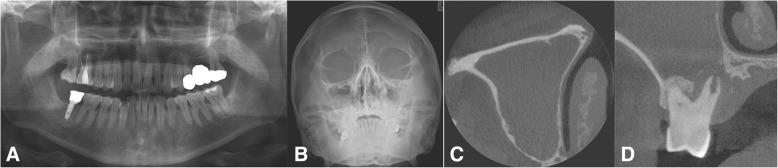
A representative case of odontogenic maxillary sinusitis originating from an apical lesion in the right upper second molar. Preoperative panoramic (**a**), Water’s (**b**), axial cone-beam CT scan (**c**), and coronal cone-beam CT scan (**d**) views

**Fig. 7:**
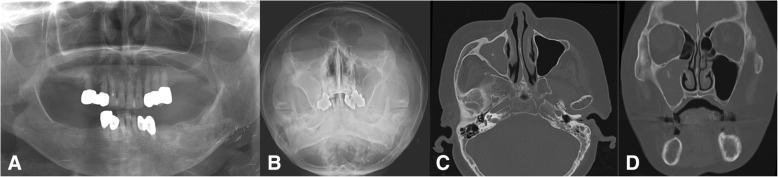
Chronic maxillary sinusitis originating from the right second premolar, the crown of which was analyzed or continuous denture loading. Preoperative panoramic view (**a**), Water’s view (**b**), and axial CT scan view showing radiopacity in the middle of the sinusitis suspicious of fungal ball (**c**), and coronal CT scan view showing definite fungal maxillary sinusitis with ostium obstruction (**d**)

**Fig. 8:**
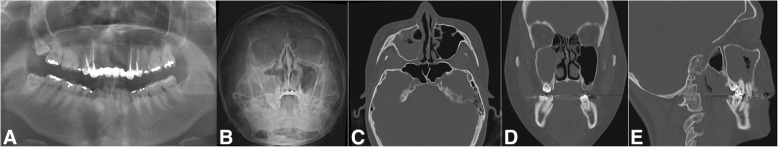
Odontogenic maxillary sinusitis originating from both an impacted third molar and a root infection of an external resorbed first molar in the right maxillary sinus. Preoperative panoramic view (**a**), Water’s view (**b**), and axial CT scan view showing an air-bubble and including a sinus mass (**c**). A coronal CT scan view showing the direct involvement of the right first molar (**d**) and a sagittal CT scan view showing direct involvement with three molars (**e**)

A variety of odontogenic diseases involve the maxillary sinus, from the lining of the sinus to the adjacent paranasal sinuses and dental tissues, or from the adjacent bone with expansion into the sinus (Fig. 3). Tooth extraction-related OMS is the most common cause (Fig. 4), alongside other dento-alveolar lesions including dentigerous cysts (Fig. 5), radicular lesions (Fig. 6), dental caries (Fig. 7), impacted teeth, and root infections of external resorbed molars (Fig. 8). The molar region has a frequency of involvement of 47.68%, followed by the first molar (22.51%), the third molar (17.21%), and the second molar (3.97%). The premolar region is involved in 5.96%, followed by the canine in 0.66% [20, 21].

Otorhinolaryngologists and medicinists have defined OMS as an iatrogenic dental disease, but these explanations are inaccurate due to deficiencies in knowledge regarding the anatomy and physiology of the maxillary sinus. Oroantral fistula (OAF) with or without tooth extraction, retained root infections, periodontitis, and other related odontogenic pathologies (Fig. 3) are the most common etiologies of OMS. Inferior maxillary sinus floor elevation after bone graft procedures, sinus floor perforation or poor positioning during dental implant fixture installation, extruded endodontic obstructive materials, foreign bodies present after apicoectomy, and surgical extraction of an impacted third molar may all be considered iatrogenic causes of OMS. However, all these procedures can be made safer when clinicians use safe, accurate approaches, even in patients with severely pneumatized maxillary sinuses.

#### Diagnosis of OMS

The most frequent clinical features of OMS can be divided into dental and sinonasal symptoms. Dental symptoms including involved tooth pain and hypersensitivity are not easily identified as odontogenic causes, but infrequent dental discomfort may occur after OMU patency preservation with continuous progression of maxillary sinus symptoms [11, 22, 23]. Representative sinonasal symptoms are unilateral cheek pain with nasal obstruction, purulent rhinorrhea, foul odor, foul taste, headaches, anterior maxillary tenderness, and postnasal drip. These symptoms cannot be distinguished from other causes of rhinitis, nor can any typical symptom be considered predominant in OMS [20, 21].

Unilateral nasal obstruction with facial pain and pressure is also a common symptom in OMS, and foul odor with rotten taste combined with tooth pain appears to clinically differentiate CMS and OMS [22, 23]. The most common dental causes are periapical abscess, periodontal disease, post dental extraction, OAF, and undetected foreign bodies in the sinus. OMS can also develop due to maxillary osteomyelitis, radicular cysts, mechanical injury of the sinus mucosa during root canal treatment, overfilling of root canals with endodontic material, incorrectly positioned dental implants, and improperly performed sinus augmentation [24–26].

#### Pathogenesis of OMS

Excluding close anatomical relationships, which can be thought of as facilitating inflammatory spread from the maxillary molars and premolars to the inferior maxillary sinus wall, many other conditions can contribute to the pathogenesis of OMS. Endo-antral syndrome was presented as a spreading pulpal disease by Selden [27–29], characterized by pulpal disease, periapical radiolucency or lamina dura loss on radiographs, faintly radiopaque mass bulging into the sinus wall, and variable radiopacities on the inferior sinus wall. Rapid spreading of dental infections may also lead to infraorbital cellulitis, transient blindness, and even life-threatening cavernous sinus thrombosis [23, 27].

The prevalence of OMS with secondary periapical lesions is 16–65% [28–30], and its management is more complicated than cases with only primary lesions [31]. Endodontic lesions spreading into the sinus are characterized by epithelial cells surrounded by connective inflammatory tissues [32–34]. Endodontic lesions could become evoluted over time during the acute or invasive phase, as well as the chronic phase. The acute phase is much more invasive and can cause the spread of bacteria directly into the sinus cavity and SM, causing hypertrophic reactions. Furthermore, if endodontic treatment does not eliminate the causative microorganisms, these hypertrophic reactions can lead to recurrent periodontitis or secondary periapical lesions [31, 35].

Other causes of OMS are SM mucosal edema with inflammatory cell infiltrates, odontogenic or mucous retention cystic formation, hypertrophic scarring or granulation, hyalinization, and necrotic odontogenic infections [14]. Apical lesions may lead to inflammation and thickening of the SM adjacent to the involved tooth roots and consequently to periapical osteitis with sinus mucosal hyperplasia [36, 37].

#### Microbiology and biofilm hypotheses

OMS has basic polymicrobial characteristics, with predominantly anaerobic bacteria in both the oral cavity and upper respiratory tract. Aerobic *Staphylococcus aureus* and *Streptococcus pneumonia* (*S. pneumonia*) and anaerobic *Peptostreptococcus* and *Prevotella* spp. are found in more than 75% of cases, while methicillin-resistant *Staphylococcus aureus* is found in 10–12% of OMS patients [38, 39]. Intraradicular bacterial and fungal genera and species such as *Streptococcus*, *Propionibacterium*, and *Candida albicans* may cause secondary periapical lesions, and more than 158 bacterial species and 3 fungal species may be also involved in the etiology of secondary periapical infections with the most common being *Enterococcus faecalis* bacteria [40, 41].

OMS-related periapical lesions have biofilm granules related to granulomatous lesions [42]. The bacterial biofilm (BB) hypothesis of OMS was recently proposed, implicating dynamic polymicrobial communities with slow replicating strains embedded in the extracellular polymeric matrix including exopolysaccharides, proteins, and nucleic acids [38]. These matrix substances are arranged in discrete layers between metabolically active strains in active outer coatings exposed to higher oxygen and nutrient concentrations, with quiescent bacteria in the deeper and inactive anaerobic core [39]. Deeper layers are relatively protected from antibiotics, detergents, and other antimicrobial compounds under humoral or cellular immunity [39], thus making them responsible for recalcitrant chronic infections.

BB has a detection rate of 70% in 25–100% of CRS samples [43–47]. BB may also act as a mechanism in OMS with chronic paranasal sinus inflammation and respiratory mucosal biofilm [48, 49]. The main pathogens involved in OMS BB are *S. aureus*, *Haemophilus influenza* (*H. influenza*), *P. aeruginosa* (*P. aeruginosa*), coagulase-negative staphylococci, *Moraxella catarrhalis*, *S. pneumoniae*, and fungal species [40, 41], as well as anaerobic species. Sometimes, displaced implants or endodontic materials inside the maxillary sinus do not result in signs of maxillary sinusitis, despite the fact that odontogenic infections are the cause of maxillary sinusitis in most OMS cases. Excluding concomitant nasal conditions including OMU status, the presence of BB should be determined when OMS develops [43, 44, 50, 51].

##### Actinomyces in the maxillary sinus

*Actinomyces* spp., including *A. israelii* and *A. radicidentis*, can be found on extraradicular granules inside the maxillary sinus due to their peculiar surface structures that allow epithelial attachment to inflammatory cells and oral bacteria [52, 53]. The extraradicular lesions caused by actinomycosis are resistant to host immune system responses, antibiotics, and orthograde treatment because orthograde endodontic treatment by itself does not reach the extraradicular bacteria [26]. The difficulty of treating *Actinomyces*-involved maxillary sinus infections indicates that alternative means of treating apical periodontitis, or apical surgery, may be required for the successful management of *Actinomyces*-related OMS [24]. Despite difficulty in distinguishing apical periodontitis caused by extraradicular or intraradicular microorganisms based on clinical signs and radiography, actinomycosis might be considered to be related to specific clinical signs and symptoms of OMS [25].

##### Fungal sinusitis

**Fig. 9:**
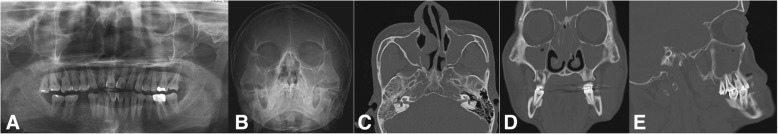
Pan-sinusitis on both paranasal sinuses originating from the root infection of the right first molar. Preoperative panoramic view (**a**), Water’s view (**b**), axial CT scan view showing sinusitis of both maxillary sinuses (**c**), coronal CT scan view showing whole sinusitis including both ethmoidal and frontal sinuses (**d**), and sagittal CT scan view showing involvement of the root pathologic lesion of the right first molar (**e**)

In immune-compromised OMS patients, including those with poorly controlled diabetes mellitus, HIV infections, or undergoing chemotherapy, fungal infections are also seen in the maxillary sinus. Aspergillosis or mucomycosis may extend to the orbital wall, temporal fossa, and even to the brain, thus producing signs and symptoms suggestive of malignant disease [54]. Most of these fungal species are inhaled through the respiratory tract and persist in the sinus mucosa by making molds and spores. The foci of infection may lead to dystrophic calcification and the formation of rhinoliths, which may be seen on dental radiographs, with large rhinoliths known as fungal balls. When fungal infection occurs with relation to dental foreign materials, the infection is normally contained within the confines of the maxillary sinus (Fig. 9) [55].

Main treatment with surgical approaches should be considered for the radical removal of any predisposing causes and for the restoration of normal MCC function. As most of these patients will be clinically immunodeficient or hospitalized, more delicate attention is required for the identification of early signs or symptoms.

### Management of odontogenic maxillary sinusitis

#### Early diagnosis with management

Although chronic sinusitis, including CRS and OMS, is common, accurate and early diagnosis is essential for successful management. In general, CRS will not initially cause facial pain, and a dentist may miss CRS in orofacial pain patients. Initial treatment such as nasal irrigation combined with the application of nasal decongestants should proceed after establishing the presence of nasal obstructions or polyps under endoscopic examination or via CT scans. If polyps are present, topical or systemic steroids should first be prescribed, and very limited use of nasal decongestants might be recommended. For surgical considerations of CSD or recurrent sinus disease, restoration of normal MCC function and clear opening of the OMU should be demonstrated first [56]. Several abnormal conditions, such as deviated septum, blocked polyp or turbinate, increased size of ostium, and hypertrophic middle meatus tissues should be managed by an otorhinolaryngologist using endoscopic views. After this initial management, odontogenic causes should be explored by the dentist or maxillofacial surgeon.

Mucous retention cysts are frequently found on panoramic views and CT scans of the floor of the maxillary sinus and are often confused with odontogenic inflammatory cysts (Figs. 5 and 6). Despite the fact that often, no treatment is recommended, retention cysts can be easily removed with an endoscopic-assisted approach due to their enlarging and non-self-remission characteristics. Mucoceles are found frequently in the maxillary sinus and are mostly located in the frontal sinus when sinus drainage is blocked. They occur when secreted mucus collects and leads to bony expansion with a strong pressure effect [57]. CRS that occurs after receiving high-dose radiation or in patients with cystic fibrosis may also require early management due to thick mucinous secretions with recurrent scar formation [5, 6].

#### Prompt management

Unilateral continuous or recalcitrant discomfort with or without foul odor is common in OMS, but the comprehensive diagnosis of OMS by dentists is difficult. Excluding CT scans or cone-beam CT, panoramic and Water’s view can be used for the identification of sinusitis of dental origin, and thus dental management alone may be adequate to resolve OMS at first, followed by subsequent surgical approaches including FESS or CLP. Facial pain with tension headache and temporomandibular joint disorder after upper respiratory tract infection may sometimes be mistaken for OMS, but the origin of pain from the sinus or nasal cavity should be assessed for accurate discrimination [58].

**Fig. 10:**
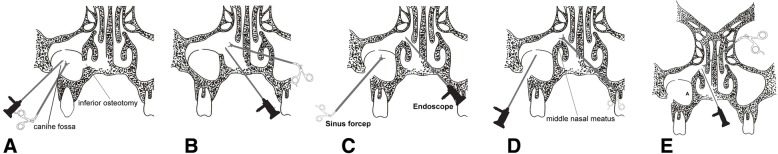
Schematic drawings of surgical approaches in odontogenic maxillary sinusitis patients showing conventional CLP (**a**), FESS (**b**), and MESS (**c**, **d**, **e**)

There are several classic surgical strategies for approaching the maxillary sinus, such as the CLP and ESS, and these methods continue to be chosen by many surgeons, although they are accompanied by many complications. CLP, confusingly termed the Caldwell Luc operation, is widely used due to easy access and quick relief of symptoms. However, two typical complications, such as the formation of postoperative maxillary cysts (POMC) and high rates of inferior osteotomy blockade, are often inevitable. In addition, high incidences of postoperative facial swelling due to intraoperative hemorrhage, facial or teeth paresthesia from infraorbital nerve involvement, and sclerosis of the maxillary sinus wall occur after classic CLP [59–61] (Fig. 10a). Furthermore, such treatment of unexpected situations makes it difficult to reconstruct the alveolar ridge for implant or prosthetic rehabilitation.

FESS has been recommended by rhinologists due to its several advantages, including wide and flexible approaches to the paranasal sinuses (PNS) without limitation to the maxillary sinus. Anatomical widening of the middle nasal meatus with whole removal of diseased tissues and pathogens could lead to the recovery of sinus function with low morbidity and preserve the inner sinus mucosa and remaining SM (Fig. 10b). FESS has gradually replaced CLP during the past several years, but is associated with complications [62]. However, the excess removal of inner physiologic tissues of the nasal cavity and incomplete solutions to odontogenic problems are deficiencies related to surgical options for OMS managements.

#### Consolidated management

Despite the development of FESS for CRS, consolidated management of OMS should ensure that the patient is infection-free without any recurrences. Complete removal of odontogenic origins, such as involved tooth extraction or apicoectomy with endodontic treatment, is essential for the prevention of OMS complications [15, 63, 64]. Additionally, due to the high frequency of OMS in the elderly, further considerations for bony reconstruction in the posterior maxillary alveolar ridge beneath the maxillary sinus are required for future prosthetic rehabilitation [15, 16].

Three main approaches can be used for the consolidated management of OMS: an intraoral approach through the originating tooth site, endoscopic approach through the nose and OMU, and intended upper maxilla approach after making bony window. Recently, modified endoscopy-assisted maxillary sinus surgery (MESS) was applied for the intraoral reduction of blowout orbital fractures [65], removal of sinus pathologies [66], and removal of migrated implants beneath the optic canal [67]. MESS is a new, innovative sinus-approach surgical procedure, which is efficient, easy, and less complicated than other sinus approaches (Fig. 10) Due to its capability to maintain sinus physiology and preserve the middle nasal meatus without causing POMC or sinus scar after CLP, OMU enlargement could be used for PNS ventilation into the nasal cavity.

#### Prevention of anticipated complications

OAF is the most common complication related to OMS. The main cause of OAF is the extraction of a maxillary posterior tooth, which accounts for more than 80% of all OAF cases [68–70]. This form of OAF is also referred to as oronasal fistula or oroantral communication, in which CRS may consequently occur via oral mucosal penetration between the posterior maxillary alveolus and the infero-lateral wall of the maxillary sinus. The main symptom of chronic non-healing OAF is purulent discharge through the fistula, especially when the patient drinks or blows through the nose from the OAF into the oral cavity or vice versa.

**Fig. 11:**
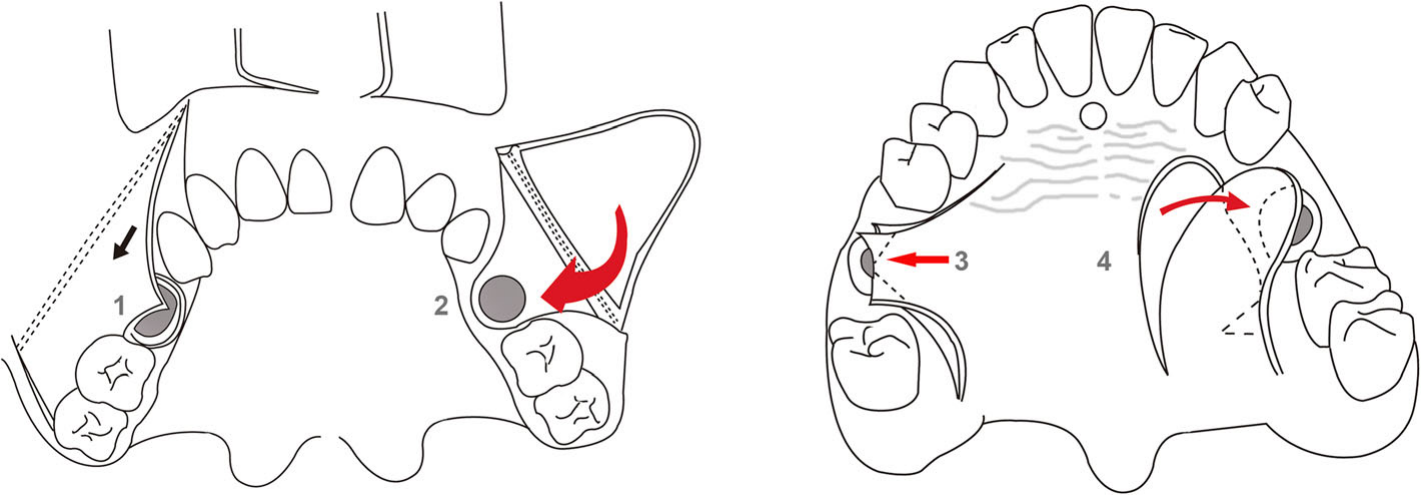
Schematic drawings of an oroantral fistula closure in the oral cavity. Direct closure (1), buccal flap (2), palatal releasing flap (3), and palatal rotational pedicled flap (4)

Regarding closure of the OAF, considerations of fistula size and depth are important for successful management. OAF can be self-covered with oral epithelium and granulation tissue or polyposis of the sinus mucosal membrane, but in cases of unsuccessful self-closure, hyperplasia of the sinus mucosal membrane can cause the formation of a very severe permanent fistula canal between the oral cavity and nose. Excluding the avoidance of OAF formation, the first solution for OAF would be CLP or FESS. The primary closure of OAF is determined according to defect size and health of the oral mucosa. Direct closure or extended surgical flaps, including buccal advancement, palatal island or pedicled flaps, may be considered for OAF management (Fig. 11). Additionally, the use of an absorbable barrier membrane, gold foils, or buccal fat pad closure could also be considered for severe OAF cases [71]. In every OAF case, maintaining a disease-free maxillary sinus membrane without infective microorganisms is also important for the functional recovery of the maxillary sinus [70–72].

## Conclusions

The incidence of OMS is much higher than previously reported and occurs in more than 30–40% of all CMS cases. Although the exact etiopathogenesis of OMS is still uncertain, common causes are known to be iatrogenic and related to dental treatment of a posterior maxillary tooth or implant procedures. An infected SM with communication to the originating dental elements may demonstrate BB formation and should be checked first. Early endoscopic and radiographic investigations by otorhinolaryngologists should be followed by dentists with intraoral diagnoses made using panoramic or Water’s views in chronic recalcitrant CRS patients. When considering treatment options for OMS, innovative approaches should be considered over conventional CLP, FESS, and MESS, due to lower rates of complications and better antral lining preservation.

## Abbreviations

ABRS: Acute bacterial rhinosinusitis
ARS: Acute rhinosinusitis
BB: Bacterial biofilm
CLP: Caldwell Luc procedure
CMS: Chronic maxillary sinusitis
CRS: Chronic rhinosinusitis
FESS: Functional endoscopic sinus surgery
*H. influenza*: *Haemophilus influenza*
MCC: Mucociliary clearance
MESS: Modified endoscopy-assisted maxillary sinus surgery
OAF: Oroantral fistula
OMS: Odontogenic maxillary sinusitis
OMU: Osteo-meatal unit
*P. aeruginosa*: *Pseudomonas aeruginosa*
PNS: Paranasal sinuses
POMC: Postoperative maxillary cysts
PRISMA: Preferred Reporting Items for Systematic reviews and Meta-Analyses
*S. aureus*: *Staphylococcus aureus*
*S. pneumonia*: *Streptococcus pneumonia*
SM: Schneiderian membrane
SRS: Subacute rhinosinusitis
URI: Upper respiratory tract infection
